# Differential *U2AF1* mutation sites, burden and co-mutation genes can predict prognosis in patients with myelodysplastic syndrome

**DOI:** 10.1038/s41598-020-74744-z

**Published:** 2020-10-29

**Authors:** Haiqiong Wang, Yongbo Guo, Zhenkun Dong, Tao Li, Xinsheng Xie, Dingming Wan, Zhongxing Jiang, Jifeng Yu, Rong Guo

**Affiliations:** 1 Department of Hematology, The First Affiliated Hospital of Zhengzhou University, Zhengzhou, 450052 China; 2 Department of Anesthesiology, The First Affiliated Hospital of Zhengzhou University, Zhengzhou, 450052 China; 3 Academy of Medical and Pharmaceutical Sciences of Zhengzhou University, #1 East Jianshe Road, Zhengzhou, 450052 China

**Keywords:** Medical research, Biomarkers, Prognostic markers

## Abstract

To investigate the *U2AF1* gene mutation site, mutation load and co-mutations genes in patients with myelodysplastic syndrome (MDS) and their effects on prognosis. Gene mutation detection by next-generation sequence and related clinical data of 234 MDS patients were retrospectively collected and analyzed for the relationship between the clinical characteristics, treatment efficacy and prognosis of *U2AF1* gene mutation. Among the 234 MDS patients, the *U2AF1* gene mutation rate was 21.7% (51 cases), and the median variant allele frequency was 39.5%. Compared with the wild type, the *U2AF1* mutant had a higher incidence of chromosome 8 aberration, and was positively correlated with the occurrence of *ASXL1*, *RUNX1*, *SETBP1* gene mutation, negatively correlated with *SF3B1, NPM1* genes mutation (*p* < 0.05). The most common mutation site of *U2AF1* was S34F (32 cases), while *U2AF1* Q157P site mutations had a higher incidence of chromosome 7 abnormalities (*p* = 0.003). The *U2AF1* gene mutation more frequently coincided with signal pathway related gene mutations (*p* = 0.043) with a trend of shortened overall survival. Among patients with *U2AF1* gene mutations, those with *ASXL1* mutations were prone to develop into acute myeloid leukemia, those with *RUNX1* mutations had an increased risk of relapse, and those with *TET2* mutations had higher 1-year survival rate. Compared with the patient group of lower mutation load (VAF ≤ 40%), the group with higher mutation load of *U2AF1* (VAF > 40%) had a significantly lower 1-year survival rate (46.1% and 80.5%, *p* = 0.027). The criteria of *U2AF1* VAF > 40% is an independent indicator for poor prognosis of MDS patients. VAF > 40% of *U2AF1* is an independent factor of short OS in MDS patients. MDS patients with a mutation in the Q157P site of *U2AF1* and a higher *U2AF1* mutation load suggests poor prognosis, and co-mutated genes in *U2AF1* can affect disease progression and prognosis.

## Introduction

Myelodysplastic syndrome (MDS) is a group of diseases with myeloid ineffective hematopoiesis, morbid hematopoiesis and high-risk transformation into acute myeloid leukemia (AML). It is characterized by malignant clonal hematopoietic stem/progenitor cell disease. About 70–90% MDS patients has one or more gene mutations^1–3^. A number of studies have confirmed that gene mutations play important role in the occurrence, development, treatment and prognosis in MDS patients^2–6^. New molecular techniques, however, such as next-generation sequencing (NGS) identifying important genetic alterations, have paved the path for new drug development targeting those specific gene mutations, and an array of new gene mutation-targeted agents are now available for MDS and AML treatment^7^. As a RNA shearing gene mutation, *U2AF1* is an early driving gene of MDS^8^. Currently, the effect of *U2AF1* on the MDS prognosis is not clear. Some studies show that *U2AF1* mutation has no effect on prognosis^9–11^, other studies show that *U2AF1* gene mutation with poor prognosis^12–15^. Studies show that *U2AF1* mutation sites may affect the prognosis. Q157 site mutation has worse prognosis than S34 mutation^14,16,17^. Gradual accumulation of mutant genes can promote the occurrence and progress of MDS. Therefore, the prognosis of MDS may be related to the accumulation of different co-mutation gene combinations^18^. In this study, gene mutation detection by NGS and related clinical data from 234 MDS patients had been analyzed to explore *U2AF1* mutation sites, mutation load and co-mutation genes of MDS and its clinical significance.

## Result

### Clinical characteristics

Among the 234 MDS patients, 142 were male (60.6%) and 92 were female (39.4%) with the median age of 55 (17–86) years old. The median number of bone marrow blasts was 6.0 (0–18.8) % at the diagnosis. The median peripheral white blood cell count (WBC) was 2.5 (0.02–20.3) × 10^9^/L; the median neutrophil (n) count was 0.95 (0–17.7) × 10^9^/L; the median hemoglobin concentration (Hgb) was 71 (24–145) g/L. The median platelet count (PLT) was 49 (3–745) × 10^9^/L. There were 32 (13.6%) patients relapsed, 36 (15.3%) developed to AML. The overall response rate (ORR) of 212 patients was 30.7% (65), and 31.6% (48) among the 134 patients with Decitabine (DEC) treatment.

### Mutant gene detection

Among the 234 patients, there were 347 mutated genes, 72.2% (169/234 patients) had at least one mutated gene, with a median mutants of 2 (1–6), while 38.0% (89/234 patients) had two or more mutated genes. 21.7% (51 patients) had *U2AF1* mutation with total of 52 mutation sites detected. Among those with *U2AF1* mutation, 84.3% (43 patients) had exon 2 mutation, 62.7% (32 patients) had *U2AF1* mutated protein sequence showing the 34th serine was substituted by phenylalanine (S34F rs371769427 , hereinafter referred as S34F), and 21.6% (11 cases) had the 34th serine was substituted by tyrosine (S34Y). 13.7% (7 cases) had the 157th glutamate was substituted by proline (Q157P). In one case, both S34F and Q157P mutations were detected at the same time. The median allele frequency (mVAF) of 51 mutation sites was 39.5% (1.38% ~ 49.02%), all of which were heterozygous mutations. Among 51 patients with *U2AF1* mutation, 39 cases (76.5%) had co-mutated genes. The common co-mutated genes were *ASXL1* (33.3%, 17 cases), *TET2* (31.4%, 16 cases), *RUNX1* (21.6%, 11 cases) and *SETBP1* (11.8%, 6 cases).

### Clinical characteristics and prognosis of *U2AF1* mutation

Compared with the non-mutated *U2AF1* group, the frequency of co-mutation between *U2AF1* gene and signal pathway associated genes of was higher in the *U2AF1* mutated patient group (22.9% and 10.6%, *p* = 0.026). The incidence of abnormal chromosome 8 (22.4% and 10.3%, *p* = 0.063) and chromosome 20 (14.6% and 7.4%, *p* = 0.161) were higher in *U2AF1* mutation group, but the difference was not statistically significant. *U2AF1* gene mutation was positively correlated with *ASXL1, RUNX1* and *SETBP1* gene mutations, but was negatively correlated with *SF3B1* gene mutation (*p* < 0.05). However, there were no significant differences in recurrence rate, risk of AML transformation, treatment response to DEC and median OS (mOS: 628 days and 790 days, *p* = 0.765) between the *U2AF1* mutation and *U2AF1* non-mutated groups;

### Clinical characteristics and prognostic significance of different *U2AF1* mutation sites

In MDS patients with *U2AF1* mutation, due to the cases of MDS-RS (0 case), MDS (5q -) (2 cases) and MDS-U (1 case) were so few, so only 48 cases of MDS-SLD/MLD/EB1/EB2 with *U2AF1* mutation were included to analyze the mutation site, mutation load and co mutation gene of *U2AF1.*

Compared to S34F and S34Y mutations, MDS patients with *U2AF1* Q157P mutation site had a significant high incidence of chromosome 7 abnormalities (*p* = 0.01), older age, relatively high risk groups with IPSS-R risk stratification, lower hemoglobin concentration, higher incidence of complex karyotypes. OS was shorter (mOS of S34F, S34Y and Q15P: 628, 804 and 284 days, respectively, *p* = 0.391). However, the difference was not statistically significant (*p* > 0.05) compared to S34F and S34Y mutations. The co-mutation rates of S34F, S34Y and Q157P amino acid mutation sites were 71.9%, 66.7% and 100%, respectively. The co-mutation frequency of two co-mutation genes for S34Y was higher than that of S34F and S34Y mutation sites. There was no significant difference for the type and proportion of co-mutation genes between S34F and S34Y loci (Table 1).

**Table 1:** *U2AF1* mutation: protein sequence and clinical characteristics.

Total	*U2AF1*mutation protein sequence [case (%)]			Total *X*^2^*/Z*	Total *p* value
	S24F	S34Y	Q157P		
	32	9	6		
Median VAF	0.4127(0.0613~0.4902)	0.397(0.0138~0.4808)	0.37215(0.0401~0.4196)		0.535
Age (year)	55(21~77)	53(33~69)	67.5(43~83)		0.088
Gender (male)	23(71.9)	8(88.9)	2(33.3)	4.854	0.091
MDS subtype 1				1.154	0.655
MDS-SLD and -MLD	12(37.5)	5(55.6)	2(33.3)		
MDS-EB1 + EB2	20(62.5)	4(44.4)	4(66.7)		
MDS subtype 2				9.350	0.097
MDS-SLD	3(9.4)	1(11.1)	2(33.3)		
MDS-MLD	9(28.1)	4(44.4)	0(0)		
MDS-EB1	7(21.9)	0(0)	3(50)		
MDS-EB2	13(40.6)	4(44.4)	1(16.7)		
IPSS-R group				1.291	0.633
Relatively low risk group	7(21.9)	2(22.2)	0(0)		
Relatively high risk group	25(78.1)	7(77.8)	6(100)		
Laboratory Results					
BM blast at diagnosis (%)	6.2(0.4~18.4)	4.8(0.4~14)	1.25(1~2.5)		0.752
N (10^9^/L)	0.77(0.06~8.63)	0.6(0.03~8.1)	1.16(0.32~6.5)		0.603
PLT (10^9^/L)	52(7~459)	38(29~394)	50(14~118)		0.86
HGB (g/L)	73.5(28~131)	102(45~145)	61.5(49~87)		0.245
Chromosomal abnormality	13(40.6)	6(66.7)	4(66.7)	2.706	0.323
Chromosome 7 abnormality	2(6.3)	0(0)	4(66.7)	11.659	0.002
Chromosome 8 abnormality	6(18.8)	4(44.4)	0(0)	3.922	0.102
Chromosome 20 abnormality	5(15.6)	2(22.2)	0(0)	1.172	0.574
Complex karyotype (≥ 3)	1(3.1)	0(0)	1(16.7)	2.577	0.275
DEC treatment response				0.913	0.738
Non-ORR	13(65)	3(60)	5(83.3)		
ORR	7(35)	2(40)	1(16.7)		
Total					
Relapse	4(12.5)	0(0)	0(0)	1.028	0.750
Transformed to AL	4(12.5)	1(11.1)	0(0)	0.538	1.000
Median OS (day)					
Co-mutation gene					
*TET2* mutation	8(25)	3(33.3)	4(66.7)	3.849	0.134
*ASXL1* mutation	13(40.6)	3(33.3)	1(16.7)	1.153	0.660
*RUNX1* mutation	8(25)	1(11.1)	1(16.7)	0.713	0.860
*TP53* mutation	1(3.1)	0(0)	1(16.7)	2.577	0.275
Epigenetic gene (+)	18(56.3)	4(44.4)	4(66.7)	0.806	0.744
Signal pathway gene (+)	6(18.8)	1(11.1)	4(66.7)	6.074	0.040
Other pathway gene (+)	10(31.3)	2(22.2)	3(50)	1.34	0.450
Number of co-mutation genes				8.396	0.160
0	9(28.1)	3(33.3)	0(0)		
1	11(34.4)	2(22.2)	1(16.7)		
2	2(6.3)	2(22.2)	3(50)		
≧ 3	10(31.3)	2(22.2)	2(33.3)		

### Clinical characteristics and prognosis of co-mutation of *U2AF1*, *ASXL1*, *TET2* and *RUNX1*

A. Compared to *U2AF1* + *ASXL1*-, patients with *U2AF1* + *ASXL1* + had a higher risk of progress to AML (29.4% and 0.0%, *p* = 0.04). There was a increasing recurrent rate (17.6% and 2.9%, *p* = 0.102). Between these two groups, there was no significant difference in IPSS-R grouping, age, gender and the response to DEC treatment and prognosis.
B. Compared to *U2AF1* + *TET2*- patients, the mutation load of *U2AF1* was lower in 16 cases of *U2AF1* + *TET2* + (mVAF: 41.3% and 35.4%, *p* = 0.034), the one year OS rate had trend of increase (one year OS: 60.3% and 66.7%, *p* = 0.109), and the ORR rate was higher in the group of DEC treatment (23.8% and 45.4%, *p* = 0.252) with no statistically significance. Among the 9 cases with mutation of *U2AF1* S34F site mutation with *TET2* + , 8 patients survived, one case lost to follow-up, suggesting that *U2AF1* patients with S34F co-mutation with *TET2* may have a better prognosis.
C. Compared to *U2AF1* + *RUNX1*-, the recurrence rate is high (30.0% and 2.6%, *p* = 0.025). However, there were no significant differences in MDS subtype distribution, IPSS-R group, age, gender, response to DEC treatment, disease progression and prognosis between the two groups.

### Clinical characteristics and prognosis of mutation load of *U2AF1* gene.

**Figure 1:**
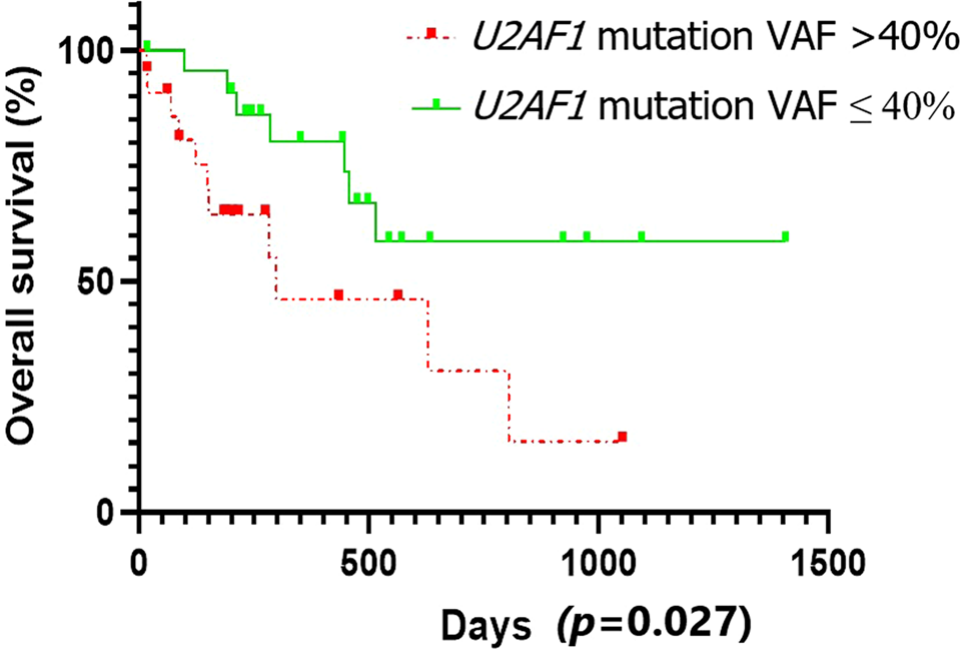
Compared with the group with relatively low mutation load (VAF ≤ 40%), the group with relatively high mutation load (VAF > 40%) has a significantly lower OS (46.1% and 80.5%, *p* = 0.027).

According to mVAF (39.5%) of *U2AF1* mutation, the patients were divided into two groups: the group with relatively low mutation load (VAF ≤ 40%) and the group with relatively high mutation load (VAF > 40%). The incidence of chromosome 7 abnormalities was lower in the group with higher *U2AF1* mutation load (0% and 23.1%, *p* = 0.025), and the 1-year survival rate was significantly lower (46.1% and 80.5%, *p* = 0.027) (Fig. 1). There was no significant difference in age, gender, response to DEC treatment, disease progress, and recurrence between the two groups.

### Gene mutation and prognosis

Among the 234 MDS patients, the median OS was 286 days (2–1406 days), with 90 (38.5%) patients died and 45 (19.2%) patients lost of follow-up. Univariate analysis results showed different factors were all correlated with a shorter OS: AML transformation (mOS: 447 days and 802 days, *p* = 0.028), chromosome 7 abnormality (-7, del(7q)) (mOS: 400 days and 830 days, *p* < 0.001), chromosome 20 abnormality (del(20q)) (mOS: 412 days and 802 days, *p* = 0.047), *EZH2* mutation (mOS: 256 days and 791 days, *p* = 0.005), *TP53* mutation (mOS: 497 days and 7 97 days, *p* = 0.023), *CBL* mutation (mOS: 158 days and 791 days, *p* = 0.038), *RUNX1* VAF > 40% (mOS: 432 days and 909 days, *p* = 0.034), *U2AF1* VAF > 40% (one-year OS rate: 46.1% and 80.5%, *p* = 0.027). Meanwhile, the age, proportion of bone marrow blasts, IPSS-R stratification and MDS subtype were included in the multivariate COX analysis. The results showed that VAF of *U2AF1* > 40% [HR = 3.261, 95% CI (1.654–6.430), *p* = 0.001], chromosome 7 abnormality [HR = 3.885, 95% CI (1.527–5.148), *p* < 0.001], *EZH2* mutation [HR = 8.523, 95% CI (2.558–28.395), *p* < 0.001], *TP53* mutation [HR = 4.532, 95% CI (1.885–10.895), *p* = 0.001], *CBL* mutation [HR = 4.286, 95% CI (1.294–14.193), *p* = 0.017] were independent adverse factors of OS (Table 2).

**Table 2:** MDS patients multivariate analysis.

Factors	HR	95%CI	*p* value
Chromosome 7 abnormality	3.885	2.117~7.131	< 0.001
*EZH2* mutation	8.523	2.558~28.395	< 0.001
*TP53* mutation	4.532	1.885~10.895	0.001
*CBL* mutation	4.286	1.294~14.193	0.017
*U2AF1* VAF > 40%	3.261	1.654~6.430	0.001

## Discussion

*U2AF1* gene is an RNA shearing related gene, which is the early mutation gene of MDS^8^. The mutation rate of *U2AF1* gene in MDS patients is about 5% to 20%^9,14,16,19^. Studies have shown that in MDS patients, the mutation rate of *U2AF1* in Asians is higher than that in Caucasians, and there is a significant correlation between *U2AF1* mutation and chromosome 8 abnormality (trisomy 8) in Asians^9^. In this study, the mutation rate of *U2AF1* in 234 MDS patients was 21.7%, higher than that in previous studies^9,17^. Compared with those without *U2AF1* mutation, the incidence of abnormal chromosome 8 and chromosome 20 in *U2AF1* mutant increased, but the difference was not significant; Among the 51 patients with *U2AF1* mutation, the mutation sites were all with S34 and Q157, and the mutation rate of S34F had the highest rate, similar to that in previous studies^9,17^. In other studies, Q157 mutation rate was the highest in patients with primary myelofibrosis and AML^17,20^. In addition, previous studies^9,14,17^ demonstrated that *U2AF1* mutant is prone to anemia and thrombocytopenia, and S34F mutant of *U2AF1*^21^ can have reduced erythropoiesis and abnormal granulo-monocyte differentiation via regulating the downstream target gene *H2AFY* and *STRAP* to form abnormal splicing. This study showed that the number of neutrophils in *U2AF1* mutant is low, but there is no significant difference among the three mutation sites. Due to the sample size, further verification is needed.

Recent studies have shown that specific mutations are closely related to cytogenetic abnormalities and may promote the occurrence and development of leukemia through unique pathogenesis. Kim et al.^6^ showed the S34Y site mutation of *U2AF1* was significantly correlated to chromosome 8 abnormality. The S34F site mutation was correlated to chromosome 20 abnormality, and the Q157 mutation had a high incidence of chromosome 7 abnormality, but there was no statistical difference.

In this study we observed at the first time that compared with S34F and S34Y mutants, Q157P mutation had significantly higher incidence of chromosome 7 abnormalities (*p* = 0.002), higher co-mutation rate of signaling pathway related genes (*p* = 0.004). There were also higher incidence of co-mutation with TP53, higher risk of AML transformation, and shorter overall survival time in the Q157P mutation group. However, there was no significant difference between the two groups, which was probably related to the small sample size. Zhang et al.^22^ have shown that *U2AF1* mutation can affect the expression of p53 signaling pathway and MAPK signaling pathway proteins by down-regulating the transcription of related genes. Therefore, we speculate that Q157P mutation of *U2AF1* may lead to genetic instability and increase the incidence of some specific pathogenic genes by down-regulating the transcription of related genes, thus affecting the progress and prognosis of the disease.

Li et al.^18^ showed that the clonal patterns with initial mutations (*ASXL1*, DNMT3A and *TET2*) promoted the occurrence of MDS, while the some additional driver mutations (*SF3B1*, *U2AF1* or *RUNX1*) played roles to keep the basic disease features, or give rise to different phenotypes (*BCOR*, *EZH2* or *TP53*) in individual patients. These mutations were identified as last events for MDS development to be AML. Last mutations can exist at MDS diagnosis, or emerge at AML transformation, and involve a small group of genes.

Single-allele *CEBPA* mutations and diverse *TP53* mutations were checked as the most common last event mutations. Considering the necessity of last event mutations and limited gene involvement in AML transformations, it is possible to validate a small group of last events involved mutations to develop some new strategies to block MDS progression. It is suggested that the occurrence and development of MDS is the process of gene mutation accumulation, and the different forms of co-mutation gene may have different effect for disease prognosis. At present, most of the researches focus on *U2AF1* mutation status, mutation sites, clinical characteristics and prognostic impact of the combined abnormalities of cytogenetics, and less on the clinical characteristics and prognostic impact of *U2AF1* and other co-mutation genes. In this study, the clinical characteristics and prognosis of patients with *U2AF1* mutation co-mutated with *ASXL1*, *TET2* and *RUNX1* mutations were compared with those without related gene mutations, respectively, in order to elucidate the role of co-mutation genes in patients with *U2AF1* gene mutation. The results demonstrated that *U2AF1* mutation was positively correlated with *ASXL1*, *RUNX1* and *SETBP1*, and negatively correlated with *SF3B1* (*p* < 0.05), consistent with previous studies^9,14,16,17^. Different mutation sites may have fixed co-mutation patterns. In this study, we found that the co-mutation ratio of Q157P and signal pathway related genes was significantly higher than that of other sites. In addition, the incidence of *TP53* co-mutation at Q15P was higher than that at S34, which was consistent with the study by Tefferi et al.^17^. This study also showed that the co-mutation rate of Q157 with *TP53*, *ASXL1*, IDH1, *SETBP1* was higher than that of S34. Hou et al.^23^ showed that in MDS patients with *U2AF1* mutation, those with *RUNX1* mutation were independent adverse factors affecting hematopoietic stem cell transplantation, suggesting that the type of co-mutation gene may have an impact on prognosis. In this study, patients with *U2AF1* mutation had a higher recurrence rate than those without *RUNX1* mutation. We considered that it might be related to the higher mutation load of *U2AF1* in this study. At the same time, we found that compared with those without *ASXL1* mutation, those with *ASXL1* mutation had a higher mutation load of *U2AF1* (*p* = 0.674), and the proportion of bone marrow blasts was higher (*p* = 0.145), which was more distributed in patients with MDS-EB (*p* = 0.092). The risk of transformation to AML is higher (*p* = 0.004) and the recurrence rate is higher (*p* = 0.121). However, the mutation load of *U2AF1* in *TET2* mutation group is lower than that in *TET2* non-mutation group (41.3% and 35.4%, *p* = 0.034), and the one-year OS rate increases (60.3% and 66.7%, *p* = 0.109). The ORR rate with DEC treatment is higher (33.3% and 30.0%), and the difference is not statistically significant. This study found that the mutation load of *U2AF1* in different co-mutation gene combinations may affect the mutation load of *U2AF1*. The mutation load may have certain effect on disease progression and prognosis. In this study, the number of cases of *U2AF1* and *TET2, ASXL1, RUNX1* co -mutation were 16, 17 and 10, respectively, with a small sample size. Therefore, the clinical data and survival analysis were not conducted according to the mutation load of the co-mutation genes. The sample size will be further expanded in the future to study the impact of each co-mutation gene load in MDS patients with *U2AF1* mutation.

Currently, the effect of *U2AF1* mutation on prognosis is not clear yet. Some studies showed that *U2AF1* mutation has no effect on prognosis 9–11), and others show that the prognosis of patients with *U2AF1* mutation is poor 12–15. Recent studies have shown that there are differences in prognosis among different *U2AF1* mutation sites. The prognosis of patients with *U2AF1* Q157 mutant is worse than that of S34^14,16,17^. In this study, the OS of Q157P has a shorter OS, compared with other loci (S34F, S34Y and mOS of Q157P: 628 days, 804 days and 284 days, *p* = 0.418), but the difference is not significant. Further validation is needed with a large sample study. In addition, some studies have shown that the mutation load of *TET2* and *TP53* has an effect on the overall survival time^24–26^. At present, few studies have reported that the mutation load of *U2AF1* has an effect on the prognosis. In this study, we found that compared with the group with relatively low mutation load of *U2AF1* (VAF ≤ 40%), the group with relatively high mutation load of *U2AF1* (VAF > 40%) has a significantly lower one-year survival rate (49.1% and 80.5%, *p* = 0.027). There was no significant difference in age, gender, response to DEC treatment, disease progression and recurrence between the two groups. The poor prognosis may be related to the higher incidence of *RUNX1* mutation (31.8% and 11.5%, *p* = 0.152). In previous studies, the patients with *RUNX1* co-mutation are more likely to progress to AML with poor prognosis^27,28^. In this study, multivariate analysis confirmed for the first time that VAF > 40% of *U2AF1* was an independent impact factor of MDS patients with a shorter OS. It has further verified the influence of *U2AF1* mutation load on disease progression and prognosis when *U2AF1* co-mutated with *TET2*, *ASXL1* and *RUNX1*. However, more studies are needed to explore the relationship between co-mutation gene and mutation load, as well as its role in disease progression and prognosis.

In summary, our results show that VAF > 40% of *U2AF1* is an independent factor of short OS in MDS patients, and the OS of MDS patients with Q157 mutation of *U2AF1* was shortened, but the difference was not significant, which needs to be further verified in the future.

## Materials and methods

### Patient cohort

From January 2016 to January 2020, data from 234 MDS patients were collected retrospectively. Informed consent was obtained from all patients or legal guardians and the study protocol was approved by the Ethics Committee of the First Affiliated Hospital of Zhengzhou University and based on the ethical principles for medical research involving human subjects of the Helsinki Declaration. Diagnosis was done according to the standard diagnosis with MICM criteria based on the 2016 WHO MDS diagnosis and classification criteria^29^. Inclusion criteria:

- De novo MDS patients diagnosed according to the WHO (2016) MDS diagnosis and classification criteria;
- Tested with NGS at the first diagnosis;
- Older than 16 years old.

According to the MDS diagnosis criteria, 17 Cases (7.3%) patients diagnosed with MDS with single-lineage dysplasia (MDS-SLD), 52 cases (22.2%) patients diagnosed with MDS with multi-lineage dysplasia (MDS-MLD), 51 cases (21.8%) diagnosed with MDS with excess blasts subtype 1 (MDS-EB1), 98 cases (41.9%) diagnosed with MDS with excess blasts subtype 2 (MDS-EB2), 10 cases (4.3%) MDS with ring sideroblasts (MDS-RS), 4 cases (1.7%) MDS with isolated del (5q) and 2 cases (0.9%) with MDS unclassifiable (MDS-U), respectively. According to the revised international prognostic scoring system (IPSS-R), 205 MDS patients with chromosomal karyotype were stratified by IPSS-R. There were 54 cases in the intermediate-risk group (26.3%), and 70 cases in the high-risk group (34.2%) and 43 cases in the very-high risk group (23.9%). According to the prognosis score system, MDS patients were divided into relatively low-risk group (IPSS-R score ≤ 3.5) and relatively high-risk group (IPSS-R score > 3.5), with 47 cases (22.9%) and 158 cases (77.1%), respectively.

### Next generation sequence

PCR was used to amplify and sequence genes. Gene mutation detection was done with standard NGS technology on a Illumina MiSeq System (Illumina, San Diego, CA) high-throughput sequencing platform with Amplicon panel targeting complete coding exons and their adjacent splice junctions from 22 genes was designed using Illumina Design Studio software v6.0 (https://designstudio.illumina.com/Home/SelectAssay) . Details of the variant calling, filtering, and annotation are described in our recently published reports^2,30,31^. Analyses were conducted of the relevant mutations of 22 related genes, including *FLT3-ITD, NPM1, KIT, CEBPA, DNMT3A, IDH1, IDH2, TET2, EZH2, RUNX1, ASXL1, PHF6, TP53, SF3B1, SRSF2, U2AF1, ZRSR2, NRAS, CBL, SETBP1, ETV6,* and *JAK2*. For the gene length longer than 150 bp, such as *FLT3-ITD*, alternative RT-PCR method was used for analysis. The pathogenic mutation sites were determined according to the COSMIC database and literature reports 30, 31).

### Treatment methods

Of the 234 MDS patients, 212 were treated with different regimen. 67 were treated with demethylation agent (HMA), 85 were treated with combination of HMA and CAG regimen, 60 patients were treated with other treatment groups (including supportive treatment and chemotherapy alone). Total of 152 patients were treated with HMA, of which 13 were treated with DEC and Azacytidine (AZA), 5 with AZA alone, 134 with DEC alone. Among the 134 patients treated with DEC, there were 60 patients with low dose (6-8 mg/m2/d) and 74 patients with high dose (12-15 mg/m2/d) of DEC. In addition, according to whether they were treated with chemotherapy, 58 patients were classified into the DEC treatment alone group and 76 patients into the DEC + other agents’ chemotherapy group.

### Treatment and efficacy criteria

Based on the efficacy criteria of the International Working Group on myelodysplastic syndrome (IWG), the ORR was defined as the sum of complete remission (CR), complete remission of bone marrow (MCR), partial remission (PR) and hematological improvement (HI) after 2–6 courses of drug treatment.

### Follow up

In this study, the medical records and telephone follow-up methods were used for both hospitalized and clinic visited patients. The starting point of follow-up was the date of diagnosis of MDS, and the end point of follow-up was either of the deadlines of follow-up (2020.2.1), the time of death or the time of loss of follow-up (LOF). As of the follow-up date, the mOS of 234 MDS patients was 189 (2–1406) days, including 99 survivals (42.3%), 90 deaths (38.5%) and 45 LOF (19.2%). Overall survival (OS) is defined as the time from start of randomization to death for any reason. If the patient is still alive by the end of the follow-up date, then the end time of total survival period is the end time of follow-up (2020.2.1).

### Statistical analysis

SPSS 26.0 software was used for statistical analysis, and chi square test or Fisher exact probability methods were used for counting data, non-parameter test was used for measurement and grade data. Spearman correlation analysis was used for non-normal distribution data. Logistic regression analysis was used for multivariate analysis. Kaplan–Meier method was used for survival analysis. Log rank test was used for comparison of differences between groups. Cox regression analysis was used for significant indicators in survival analysis. P < 0.05 was considered as statistically significance.

### Ethics approval and consent to participate

Informed consent was obtained from all patients or legal guardians and the study protocol was approved by the Ethics Committee of the First Affiliated Hospital of Zhengzhou University and based on the ethical principles for medical research involving human subjects of the Helsinki Declaration.

## Consent for publication

All authors agreed to publish.

## Data Availability

Data and material will be available upon corresponding author approval. All data sets generated/analyzed for this study are included in the manuscript and the additional files.

## Abbreviations

AML: Acute myeloid leukemia
AZA: Azacytidine
CR: Complete remission
DEC: Decitabine
HI: Hematologic improvement
HMA: Hypomethylating agent
IPSS-R: Revised international prognostic scoring system
IWG: International Working Group
MCR: Complete remission of bone marrow
MDS: Myelodysplastic syndrome
MDS (5q-): MDS with isolated del (5q)
MDS-EB1: MDS with excess blasts subtype 1
MDS-EB2: MDS with excess blasts subtype 2
MDS-MLD: MDS with multi-lineage dysplasia
MDS-RS: MDS with ring sideroblasts
MDS-SLD: MDS with single-lineage dysplasia
MDS-U: MDS unclassifiable
mOS: Median overall survival
mVAF: Median variant allele frequency
NGS: Next-generation sequence
ORR: Overall response rate
OS: Overall survival
PR: Partial remission
VAF: Variant allele frequency
WBC: White blood cell count
